# Immune checkpoint inhibitor-induced nephrotic syndrome: a pharmacovigilance analysis of 404 FAERS cases and literature case series

**DOI:** 10.1080/0886022X.2025.2569089

**Published:** 2025-10-14

**Authors:** Jingjing Ye, Jiangfeng Huang, Leping Shao, Zhi Wang

**Affiliations:** ^a^ The First Affiliated Hospital of Xiamen University, School of Medicine, Xiamen University, Xiamen, China; ^b^ Department of Nephrology, (Fujian Provincial Clinical Research Center for Glomerular Nephritis) The First Affiliated Hospital of Xiamen University, School of Medicine, Xiamen University, Xiamen, China

**Keywords:** Immune checkpoint inhibitor, nephrotic syndrome, pharmacovigilance, immune-related adverse events, oncology, FAERS database

## Abstract

Immune checkpoint inhibitor (ICI)-related nephrotic syndrome is a rare but increasingly reported adverse event, where early-onset severe cases warrant heightened vigilance. This pharmacovigilance study assessed this correlation by analyzing the U.S. Food and Drug Administration’s Adverse Event Reporting System (FAERS) database from Q1 2011 to Q1 2025. A total of 404 cases of ICI-related nephrotic syndrome were identified in the FAERS database, with 84.1% of cases related to programmed cell death protein 1 (58.9%) or programmed death-ligand 1 (25.2%) inhibitors. Disproportionality signals were observed for six ICI monotherapies and one combination therapy: atezolizumab (ROR 6.84; 95% CI: 5.58–8.40), avelumab (ROR 5.54; 95% CI: 2.64–11.63), nivolumab (ROR 4.37; 95% CI: 3.64–5.25), pembrolizumab (ROR 3.44; 95% CI: 2.88–4.10), durvalumab (ROR 2.03; 95% CI: 1.12–3.66), ipilimumab (ROR 2.05; 95% CI: 1.07–3.94), and the combination therapy of nivolumab and ipilimumab (ROR 4.56; 95% CI: 3.56–5.83). Firth multivariate logistic regression analysis identified several independent risk factors, including advanced age (>65 years), malignant renal neoplasm, malignant mesothelioma, and the concomitant use of bevacizumab or lenvatinib. The median onset time was significantly shorter for severe cases than for non-severe cases (22.5 vs. 96.5 days, respectively; *p* = 0.0479). Additionally, a literature review of 18 cases provided supplementary information on the clinical features. This study provides vital pharmacovigilance insights regarding nephrotic syndrome related to ICIs, enhancing the understanding of its clinical implications.

## Introduction

Since the first immune checkpoint inhibitor (ICI), ipilimumab, was approved in 2011, ICI-based therapies have fundamentally transformed the landscape of cancer treatment over the past decade, demonstrating significant clinical success in various malignancies [1–3]. These agents exert antitumor activity by inhibiting checkpoints of the immune system, including cytotoxic T-lymphocyte–associated antigen 4 (CTLA-4), programmed cell death protein 1 (PD-1), and programmed death-ligand 1 (PD-L1) [4,5]. However, with widespread use of these novel therapies, a spectrum of immune-related adverse events (irAEs) has emerged. The overall incidence of irAEs among patients receiving ICI therapy has been reported to range from 15 to 90%, encompassing manifestations such as rash, arthritis, endocrinopathies, colitis, and pneumonitis [6,7]. The clinical features and management of these common irAEs have been thoroughly summarized in several authoritative reviews [8,9].

Regarding kidneys, acute interstitial nephritis (AIN) is the most widely known irAE, with its diagnosis, treatment, and prognosis having been well described [10–13]. However, nephrotic syndrome, with its relatively lower incidence, is often overlooked. Nephrotic syndrome is characterized by heavy proteinuria, hypoalbuminemia, and dyslipidemia caused by disruption of the glomerular filtration barrier [14]. However, similar symptoms can also present in patients with advanced tumors, this increases the difficulty in the clinical diagnosis of ICI-related nephrotic syndrome [15]. Prompt withdrawal of the culprit drug is the core of treatment in any drug-induced kidney disease. Therefore, it is critical to understand the occurrence of nephrotic syndrome with ICIs for clinical practice.

To date, reports on ICI-related nephrotic syndrome remain exceedingly limited, primarily restricted to case reports [16–31]. Consequently, little is known regarding its onset time, risk factors, and outcomes. The U.S. Food and Drug Administration’s (FDA) Adverse Event Reporting System (FAERS) is one of the largest individual case safety report (ICSR) databases, collecting adverse drug reaction cases from clinical practice worldwide, with a special advantage in monitoring rare or delayed adverse events. Based on large-scale real-world datasets, disproportionality analysis can be employed to detect disproportionate reporting signals for this adverse event, providing a more comprehensive assessment of drug safety in real-world settings [32].

Here, we queried the FAERS database and reviewed the existing literature aimed to characterize the real-world safety profile of ICI-related nephrotic syndrome to provide evidence for clinical monitoring and management.

## Methods

### Study type and data source

This study was a retrospective, cross-sectional disproportionality analysis of ICSRs retrieved from the FAERS database, designed to evaluate the reporting association between ICIs and nephrotic syndrome.

### Data description, access, and preprocessing

The data for this study were sourced from the FAERS database, which compiles spontaneous post-marketing adverse event reports for drugs, biologics, and medical devices from around the world. We downloaded and integrated all available data from the first quarter of 2011 to the first quarter of 2025 (2011Q1–2025Q1). All adverse events were coded with version 27.1 of the Medical Dictionary for Regulatory Activities (MedDRA). In MedDRA’s hierarchical structure, terms range from the most specific to the most general: Preferred Term (PT), High Level Term (HLT), High Level Group Term (HLGT), and System Organ Class (SOC). Drug names were standardized according to the Medical Subject Headings (MeSH) of the U.S. National Library of Medicine.

To address the issue of duplicate reports in the FAERS database, we followed the FDA’s recommended deduplication procedures. Cases with identical “primaryid” values were identified as duplicates, we retained only the latest report, which contain the most complete information. Missing values for key variables such as age and sex were categorized as “unknown” for the purpose of analysis. Only reports in which ICIs were designated as the “primary suspect” (PS) or “secondary suspect” (SS) drug were included in the final analysis.

### Variables definition

#### Study population

The primary analysis of this study included all ICSRs from the FAERS database within the specified timeframe. To ensure the generalizability of the results, the primary analysis did not impose any restrictions on reporter type, concomitant medications, or indications within the study population.

#### Drug definition

The ICIs included in this study comprised four anti-PD-1 drugs (nivolumab, pembrolizumab, cemiplimab, and dostarlimab), three anti-PD-L1 drugs (atezolizumab, avelumab, and durvalumab), and one anti-CTLA-4 drug (ipilimumab), and the combination regimen of nivolumab plus ipilimumab.

#### Adverse event definition

We utilized the PTs from the MedDRA to identify target renal adverse events (nephrotic syndrome). This included: ‘Nephrotic syndrome,’ ‘Glomerulonephritis membranous,’ ‘Mesangioproliferative glomerulonephritis,’ ‘Glomerulonephritis minimal lesion,’ ‘Focal segmental glomerulosclerosis,’ and ‘Glomerulonephritis membranoproliferative.’

### Statistical methods

#### Disproportionality analysis

We conducted a disproportionality analysis using the Reporting Odds Ratio (ROR) to evaluate the potential association between ICIs and the occurrence of nephrotic syndrome [33]. ROR is a specific statistical measure used in pharmacovigilance to identify potential associations between a drug and an adverse event. It quantifies the extent to which a particular event is reported for a specific product compared to all other products. Therefore, the ROR value provided in this study should be interpreted as a measure of the strength of the signal of asymmetry, and not as a relative risk. A signal of disproportionate reporting was considered significant if the following two criteria were met: (1) the number of cases (a) was three or more, and (2) the lower limit of the 95% confidence interval (CI) for the ROR was greater than 1. The analysis was performed using a 2 × 2 contingency table, with all other drugs and all other events in the database constituting the comparator group (Supplementary Table S1).

Second, the following sensitivity analyses were performed: (a) Restricting the analysis to reports submitted only by physicians and pharmacists, as these are generally considered more reliable; (b) excluding reports involving concomitant use of known nephrotoxic agents, which were identified by reviewing the 40 most common co-administered drugs (Supplementary Tables S2 and S3), to mitigate potential confounding; and (c) Excluding reports with nephrotic syndrome listed as an indication, considering that patients might report the primary disease as an adverse event.

**Table 2.** Summary of case reports of ICI-related nephrotic syndrome reported in the literature.

Patient	Age	Sex	Malignancy	ICI	Onset time	Histological classification	Treatment	Treatment response	Vital status	ICI rechallenge
1^16^	62	M	Malignant pleural mesothelioma	Pembrolizumab (200 mg)	10 days	MCD	Prednisone (1 mg/kg/ day)	Complete renal remission	–	–
2^17^	43	M	Hodgkin Lymphoma	Pembrolizumab (10 mg/kg q2w)	4 weeks	MCD	Prednisone (2 mg/kg)	Partial renal remission	Death due to tumor progression	–
3^17^	45	M	Melanoma	Ipilimumab (10 mg/kg qw)	18 moth	MCD	Prednisone (1 mg/kg)	Renal remission	Death due to tumor progression	Nephrotic syndrome returned 4 months after ipilimumab was restarted but resolved once the drug was stopped.
4^18^	62	M	Renal cell carcinoma	Nivolumab (3 mg/kg q2w)	4 cycles	FSGS	Methylprednisolone (1000 mg/day), Mycophenolate Mofetil	Partial renal remission	Death due to tumor progression and its complications	Proteinuria returned at a 5 mg/day prednisone dose but resolved once high-dose steroids were reinstated.
5^19^	40	M	Hodgkin lymphoma	Anti-PD-1 (200 mg q2w)	30 days	MCD	Prednisone (1 mg/kg/day)	Complete renal remission	–	–
6^20^	68	M	Melanoma	Pembrolizumab (2 mg/kg q3w)	18 days	MCD	Prednisolon (100 mg qd)	Partial renal remission	–	Proteinuria returned after two doses of ipilimumab- nivolumab rechallenge.
7^21^	79	M	Lung adenocarcinoma	Pembrolizumab (200 mg q3w)	6 cycles	MCD	Prednisolone (40 mg/day)	Complete renal remission	–	Pembrolizumab was restarted with prednisolone, and nephrotic syndrome did not recur.
8^22^	75	F	Squamous cell anal carcinoma	Nivolumab (2.4 mg/kg)	2 month	MPGN	Prednisone	No renal remission	Death due to tumor progression with failure to achieve renal remission prior to death	–
9^23^	70	F	Breast cancer	Nivolumab (–)	8 months	FSGS	Steroid	Partial renal remission	Death due to tumor progression	–
10^24^	57	M	Squamous cell carcinoma of the tongue	Nivolumab (–)	1 month	MCD	Prednisolone (75 mg/day)	No renal remission	Death due to tumor progression with failure to achieve renal remission prior to death	–
11^25^	74	M	Lung adenocarcinoma	Tislelizumab (-)	10 months	MN	Methylprednisolone (60 mg/day) and rituximab	Complete renal remission	–	–
12^26^	46	F	Melanoma	Pembrolizumab (200 mg q3w)	16 months	FSGS	ARB and diuretics	Renal remission	–	–
13^27^	69	M	Lung adenocarcinoma	Nivolumab (–)	3 Months	MN	Prednisolone (0.8 mg/day/kg)	Complete renal remission	–	–
14^28^	57	M	Renal cell carcinoma	Pembrolizumab (–)	10 months	MN	Rituximab (1 g, day 1, day 14)	Renal remission	Death due to tumor progression and its complications	–
15^29^	65	M	Lung adenocarcinoma	Pembrolizumab (4 mg/kg q3w)	1 year	MN	Methylprednisolone (500 mg/day)	Renal remission	–	–
16^30^	56	M	Non-small cell lung carcinoma	Pembrolizumab (–)	12 weeks	MN	Prednisone (1 mg/kg/d), rituxima	No renal remission	Death due to tumor progression	Nephrotic syndrome recurred after pembrolizumab rechallenge but resolved once the drug was stopped and rituximab given.
17^30^	67	M	Urothelial carcinoma of the bladder	Atezolizumab (–)	12 weeks	MN	Rituximab	Renal remission	–	–
18^31^	75	M	Malignant pleural mesothelioma	nivolumab (360 mg, q3w)+ ipilimumab (1 mg/kg, q6w)	13 days	MCD	Prednisone (1 mg/kg), hemodialysis	Renal remission	–	–

Abbreviations: MCD, Minimal Change Disease; FSGS, Focal Segmental Glomerulosclerosis; MPGN, Membranoproliferative Glomerulonephritis; MN, Membranous Nephropathy. A hyphen (–) indicates unrecorded data.

Additionally, we evaluated the potential association with nephrotic syndrome for other novel antineoplastic agents approved after 2011. The specific drug classes and individual agents included in this comparison are detailed in Supplementary Table S4. The ROR algorithm was similarly used to calculate the signal strength of other novel antineoplastic agents, and a volcano plot was developed to visually compare the signal strength for nephrotic syndrome among these drugs. Each point in the plot represents a drug, with the x-axis indicating the logarithmic value of the ROR, and the *y*-axis representing the statistical significance [−log10(FDR-adjusted *p*-value)]. The color intensity of each point correlates with the logarithmic value of the number of case reports, providing an intuitive display of signal significance and reporting frequency.

#### Risk factor analysis

To identify independent risk factors, we performed a case/non-case study using multivariable Firth logistic regression. This analysis was restricted to the final dataset of reports in which an ICI was designated as a “primary suspect” or “secondary suspect” drug. Within this cohort, reports of nephrotic syndrome were defined as ‘cases,’ and all reports of other adverse events were defined as ‘non-cases’. The dependent variable was the occurrence of nephrotic syndrome (yes/no). Independent variables included age (<65 years vs. ≥65 years), gender, primary cancer type (with malignant pulmonary neoplasm as the reference category), and the five most commonly administered non-ICI antineoplastic agents. Analyses were restricted to cases with complete information on age and gender. Univariate Firth logistic regression was first used for preliminary screening, a method that can reduce estimation bias caused by rare events and data separation [34]. Variables found to be statistically significant in the univariate analysis (*p* < 0.05) were subsequently included in the multivariable model to adjust for potential confounders and identify independent risk factors. The generalized variance inflation factor (GVIF) was used to check for multicollinearity. The final model estimated adjusted odds ratios (aORs) with corresponding 95% confidence intervals, and statistical significance was defined as *p* < 0.05. To verify model stability, stratified bootstrap resampling (1,000 times) was used to assess the robustness of major effect values and their CIs. Additionally, *E*-values were calculated to assess the robustness of significant associations against potential unmeasured confounding. An *E*-value greater than 1 provides evidence of robustness, where higher values signify a more robust association [35,36].

#### Time-to-onset analysis

The time to onset (TTO) was the time between the reported start date of ICI therapy (DRUG_START_DATE) and the date of the adverse event (EVENT_DATE). We used median, interquartile range (IQR), minimum, maximum and the Weibull shape parameter (WSP) to describe its distribution. This model comprises two key parameters: a scale parameter (*α*) and a shape parameter (*β*). The dynamic trend of the hazard function was assessed using the shape parameter (*β*) and its 95% confidence interval (CI). A *β* < 1 indicates a decreasing hazard over time; *β* ≈ 1 (with the 95% CI including 1) suggests a constant hazard; and *β* > 1 indicates an increasing hazard over time [37]. Furthermore, cases resulting in death or life-threatening outcomes were defined as severe. The Mann-Whitney *U* test was used to compare the median TTO between severe and non-severe cases. *p*-value < 0.05 considered statistically significant.

#### Case series

We searched PubMed for English-language articles published before July 13, 2025. We used the following search terms: (immune checkpoint inhibitors or checkpoint inhibitor therapy or checkpoint inhibitor treatment or nivolumab or pembrolizumab or cemiplimab or dostarlimab or atezolizumab or avelumab or durvalumab or ipilimumab) and (nephrotic syndrome or glomerulonephritis membranous or mesangioproliferative glomerulonephritis or glomerulonephritis minimal lesion or focal segmental glomerulosclerosis or glomerulonephritis membranoproliferative) and (case report or case series). Inclusion criteria encompassed any case reports or series describing nephrotic syndrome during or potentially associated with ICI treatment. We collected patient information, including age, gender, ICI regimen cycle or time to onset, kidney biopsy findings, treatment, and outcomes.

This study was reported in accordance with the Strengthening the Reporting of Observational Studies in Epidemiology (STROBE) guideline [38]. Statistical analyses and figure generation were performed using R (version 4.3.3) and GraphPad Prism (version 10). The human anatomy heatmap was generated *via* the MOAHIT web tool [39].

## Result

### Descriptive analyses

**Figure 1.**
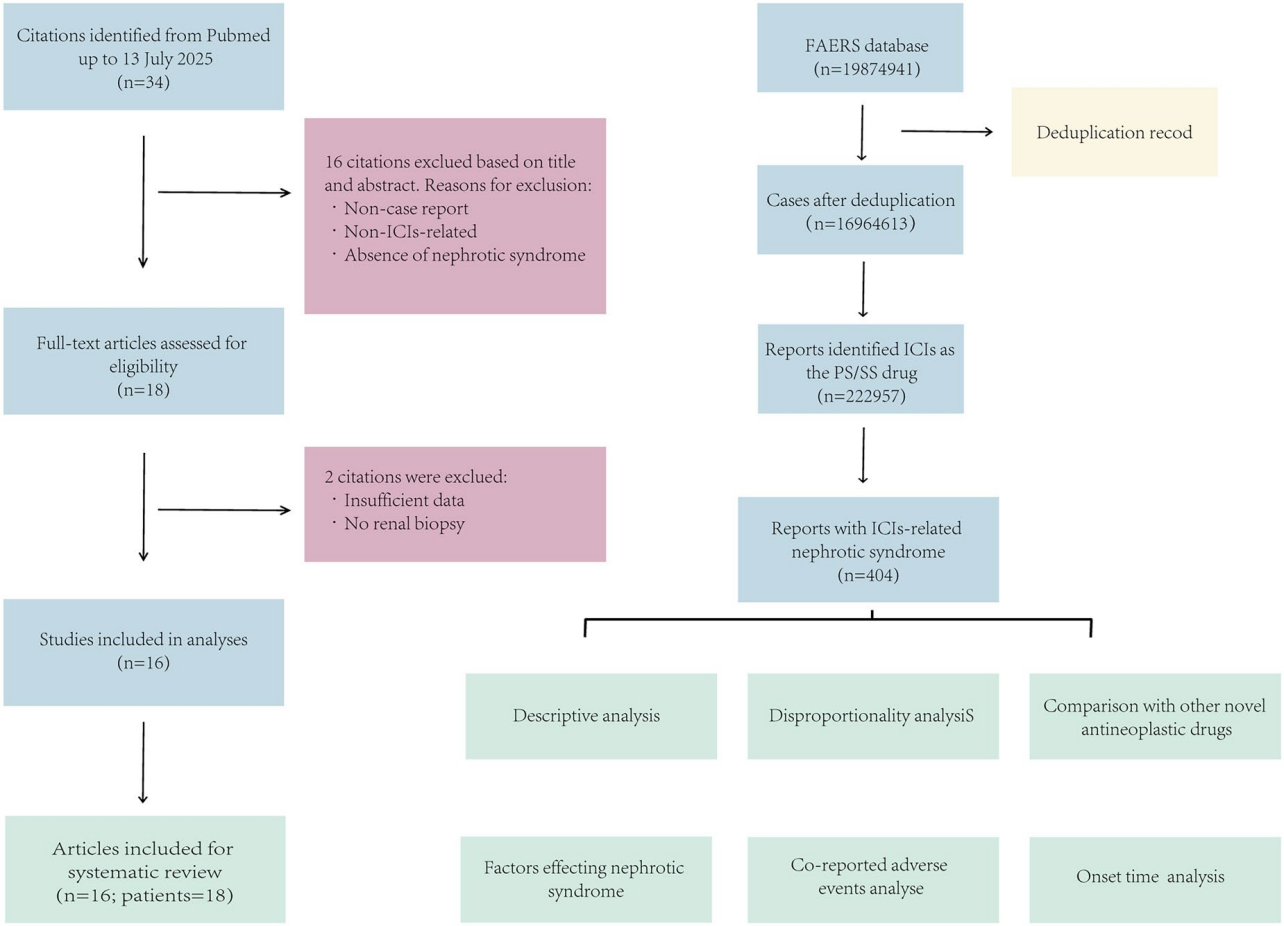
Flow chart showing the analysis process of the study.

**Figure 2.**
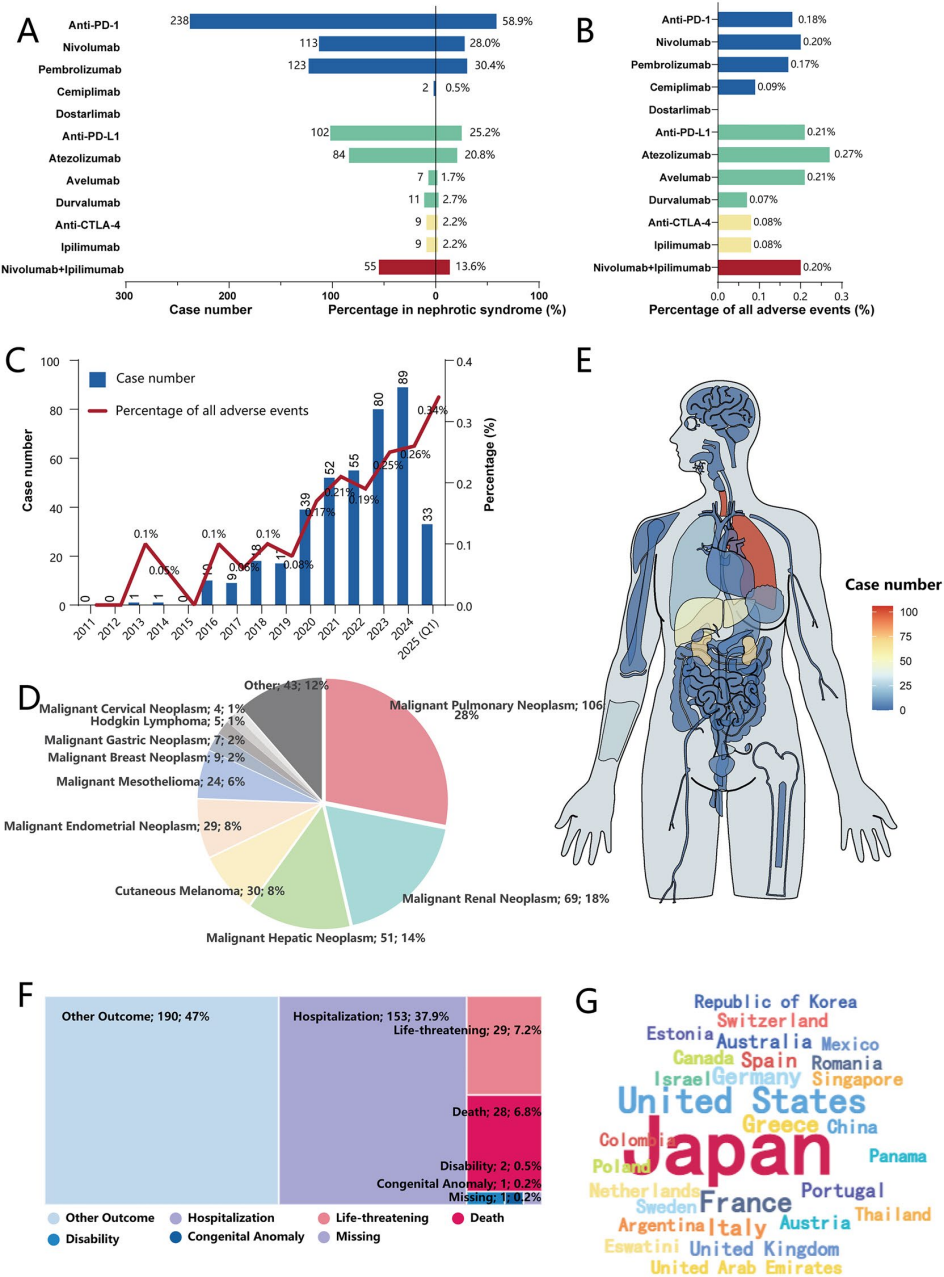
Descriptive analysis of reports with ICI-related nephrotic syndrome. (A) Distribution of nephrotic syndrome reports among ICI agents. The left panel displays the absolute number of cases for each agent. The right panel indicates the percentage of cases for each agent relative to the total number of ICI-related nephrotic syndrome reports. (B) Drug-specific reporting proportion of nephrotic syndrome. This proportion, presented as a percentage, is calculated for each agent by dividing its number of nephrotic syndrome reports by the total number of all adverse event reports associated with that same agent. (C) Annual trends in reporting of ICI-related nephrotic syndrome. The bars (left axis) show the absolute number of cases reported per year. The line (right axis) shows the annual reporting proportion, calculated as the proportion of nephrotic syndrome reports relative to all ICI adverse event reports each year. (D) Proportional distribution of the top 10 primary cancer indications for ICI-related nephrotic syndrome. The chart displays the percentage and absolute number (n) of cases for each of the most common cancer types. (E) Anatomical visualization of primary malignancies leading to ICI-related nephrotic syndrome. (F) Distribution of clinical outcomes for ICI-related nephrotic syndrome. The chart illustrates the proportion of each reported outcome, showing both the absolute number (n) and the percentage (%) of the total cases. (G) Geographic distribution of reporting countries visualized as a word cloud. The font size of each country corresponds to its total number of reported cases, with larger names indicating a higher volume of reports.

From the first quarter of 2011 to the first quarter of 2025, a total of 404 cases of nephrotic syndrome associated with ICIs were identified in the FAERS database (Figure 1). The majority of events occurred during treatment with anti-PD-1 agents (*n* = 238, 58.9%) or anti-PD-L1 agents (*n* = 102, 25.2%) (Figure 2A). The proportion of nephrotic syndrome among all reported adverse events varied by drug type: 0.08% for anti-CTLA-4, 0.18% for anti-PD-1, and 0.21% for anti-PD-L1 (Figure 2B). Trend analyses demonstrated a continuous rise, from 2011 to 2025, in both the absolute number and the relative proportion of ICI-related nephrotic-syndrome cases.

The baseline characteristics of the 404 patients are summarized in Table 1. Most patients were male (*n* = 240, 59.4%) and the median age was 69 years (IQR, 60–74 years). The majority of reports originated from healthcare professionals, principally physicians (*n* = 249, 61.6%) and pharmacists (*n* = 20, 5.0%). The leading indications for ICI therapy were malignant pulmonary tumors (*n* = 106, 28%), renal malignancies (*n* = 69, 18%), hepatic malignancies (*n* = 51, 14%) and cutaneous melanoma (*n* = 30, 8%) (Figure 2D and 2E). Clinically, 153 patients (37.9%) required hospitalization, 28 (6.9%) died and 29 (7.2%) experienced life-threatening events (Figure 2F). Geographically, Japan accounted for the largest share of cases (*n* = 199, 49.3%), followed by the United States (*n* = 58, 14.4%) (Figure 2G).

**Table 1.** The clinical characteristics of patients with ICI-related nephrotic syndrome in the FAERS database.

Characteristics	Report number (%)
Number of reports	404
Sex	
Female	121 (30%)
Male	240 (59.4%)
Unknown	43 (10.6%)
Age (years)	
<18	0 (0%)
18–64	93 (23%)
>64	201 (49.8%)
Unknown	110 (27.2%)
Median (IQR)	69 (60–74)
Outcome	
Death	28 (6.9%)
Life-threatening	29 (7.2%)
Disability	2 (0.5%)
Hospitalization	153 (37.9%)
Congenital anomaly	1 (0.2%)
Other outcome	190 (47%)
Unknown	1 (0.2%)
Type of reporter	
Physician	249 (61.6%)
Pharmacist	20 (5%)
Health professional	99 (24.5%)
Consumer	17 (4.2%)
Other reporter	17 (4.2%)
Unknown	2 (0.5%)
Reported countries (Top 3)	
Japan	199 (49.3%)
United States	58 (14.4%)
France	33 (8.2%)

### Disproportionality analyses

**Figure 3.**
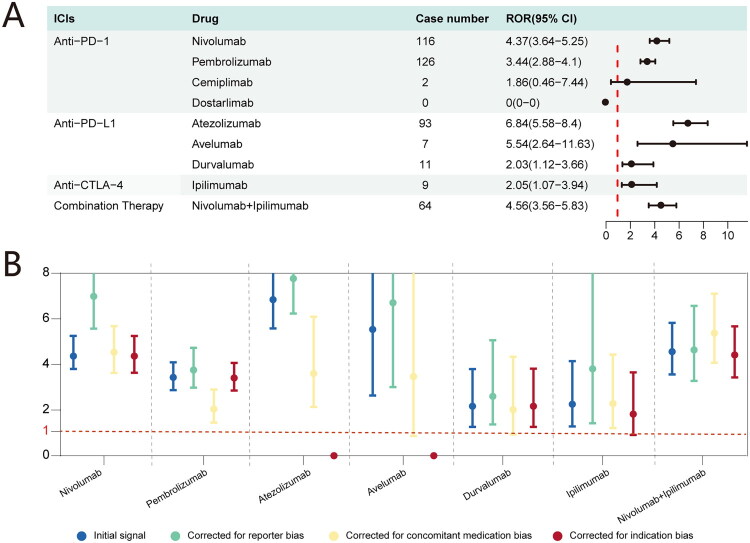
Disproportionality analysis of ICI-related nephrotic syndrome. (A) Forest plot of RORs for nephrotic syndrome associated with individual ICI agents. (B) Sensitivity analyses of the ROR for ICI-related nephrotic syndrome.

In FAERS, seven ICI regimens were identified as signal of disproportionate reporting for nephrotic syndrome (Figure 3A). The PD-L1 inhibitor atezolizumab was associated with the strongest signal (ROR 6.84; 95% CI: 5.58–8.40). Other ICIs with positive signals included the PD-1 inhibitors nivolumab (ROR 4.37; 95% CI: 3.64–5.25) and pembrolizumab (ROR 3.44; 95% CI: 2.88–4.10); the PD-L1 inhibitors avelumab (ROR 5.54; 95% CI: 2.64–11.63) and durvalumab (ROR 2.03; 95% CI: 1.12–3.66); and the CTLA-4 inhibitor ipilimumab (ROR 2.05; 95% CI: 1.07–3.94). Additionally, combination therapy with nivolumab and ipilimumab yielded a strong association signal (ROR 4.56; 95% CI: 3.56–5.83). Cemiplimab and dostarlimab were not associated with a positive signal. For cemiplimab, only two relevant reports of nephrotic syndrome were found, yielding a non-statistically significant ROR (1.86; 95% CI: 0.46–7.44). For dostarlimab, no associated reports of nephrotic syndrome were identified.

In sensitivity analyses, the signals for nivolumab, pembrolizumab, and the nivolumab-ipilimumab combination remained robust across all approaches (Figure 3B) (Supplementary Table S5). In contrast, the signals for durvalumab, ipilimumab, and avelumab showed some degree of instability when analyses were adjusted for concomitant drugs or restricted by indication. Notably, no specific indications were recorded in any of the reports involving atezolizumab or avelumab; therefore, sensitivity analyses based on indication were not applicable for these agents, suggesting that their original signals were not confounded by indication bias.

### Comparison with other novel antineoplastic agents

**Figure 4.**
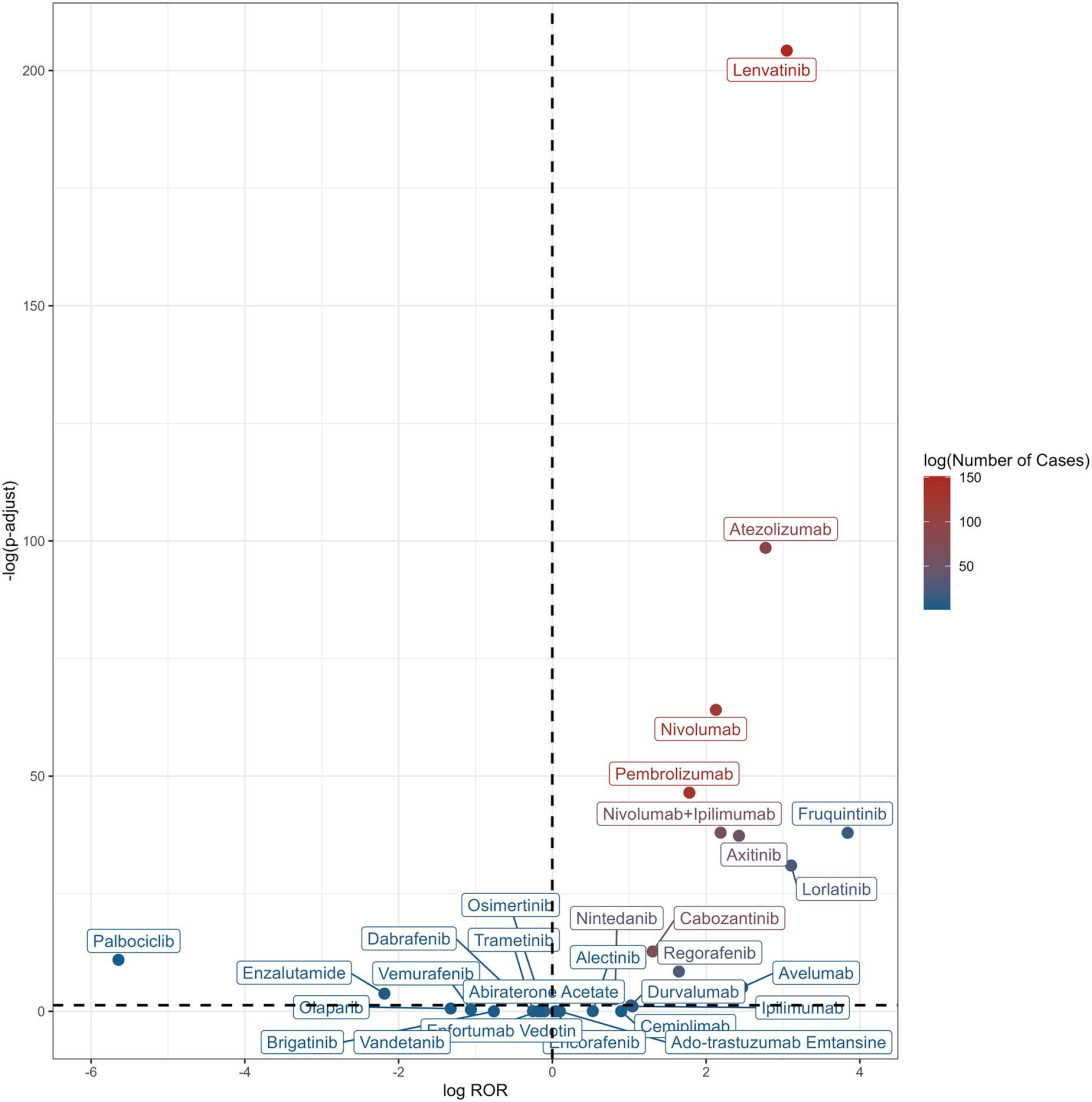
Volcano plot of nephrotic syndrome signals associated with ICIs and other novel antineoplastic agents. Each point in the plot represents a drug, with the x-axis indicating the logarithmic value of the ROR, and the y-axis indicating the negative logarithmic value of the p-value adjusted using the FDR method. The point color reflects the report count.

We also conducted a disproportionality analysis to assess the potential association of nephrotic syndrome with novel, non-ICI antineoplastic agents approved since 2011. The majority of these drugs did not yield positive nephrotic-syndrome signals; only certain anti-angiogenic agents and the ALK inhibitor lorlatinib showed an association. Overall, there are more signals of disproportionate reporting for ICIs compared with other novel antineoplastic agents (Figure 4) (Supplementary Table S6).

### Factors associated with nephrotic syndrome

**Figure 5.**
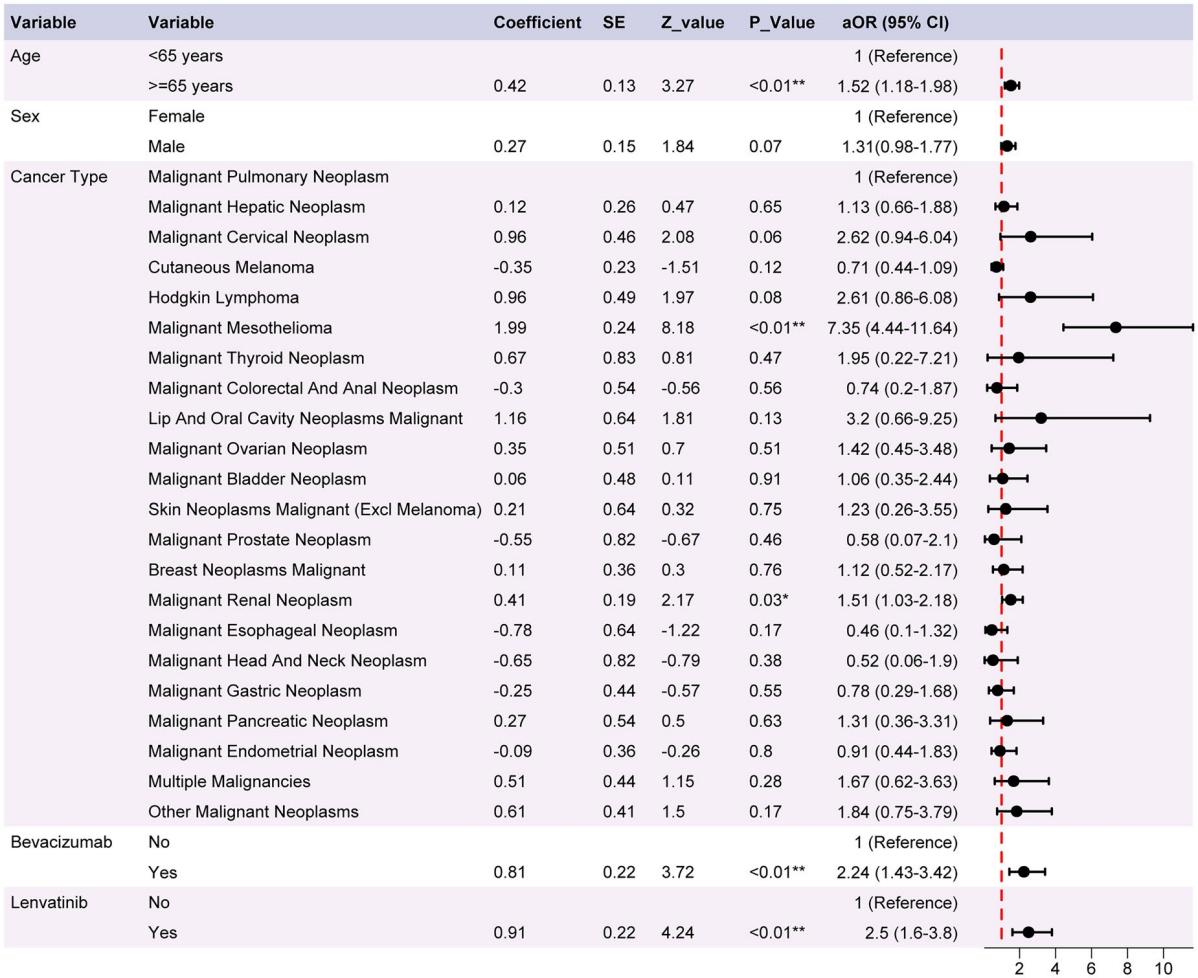
Factors independently associated with reports of ICI-related nephrotic syndrome. Forest plot showing aORs and 95% CIs from a firth multivariable logistic regression analysis. Reference categories were age <65 years, female, malignant pulmonary tumor, and nonuse for bevacizumab and lenvatinib. Asterisks indicate the level of statistical significance: ** (*p* < 0.01), * (*p* < 0.05).

Univariate Firth logistic regression analysis showed that age, sex, cancer type and the concomitant medications bevacizumab and lenvatinib were associated with the development of nephrotic syndrome (Supplementary Table S7). When the above variables were included in the multivariate Firth logistic regression model, it was found that age (> 65 years) (aOR = 1.52; 95% CI, 1.18–1.98), malignant mesothelioma (vs malignant pulmonary tumor; aOR = 7.35; 95% CI, 4.44–11.64), malignant renal neoplasm (vs malignant pulmonary tumor; aOR = 1.51; 95% CI, 1.03–2.18), bevacizumab (aOR = 2.24; 95% CI, 1.43–3.42), and lenvatinib (aOR = 2.5; 95% CI, 1.6–3.8) remained independently associated with nephrotic syndrome onset (Figure 5). Collinearity diagnostics showed corrected GVIF < 2 for all variables, indicating the absence of relevant multicollinearity (Supplementary Table S8). The 95% confidence intervals generated by the bootstrap analysis were highly consistent with those of the primary analysis, thereby reinforcing the robustness of our conclusions (Supplementary Table S9). The E-value point estimates for all significant associations were greater than 1 (Supplementary Table S10). Notably, the E-values for malignant mesothelioma (8.35), lenvatinib (2.57), and bevacizumab (2.22) were substantially greater than 1, suggesting that these associations are particularly robust against unmeasured confounding. The remaining *E*-values for age (> 65 years) and malignant renal neoplasm were more modest (1.64 and 1.21, respectively).

### Co-reported adverse events and time to onset for ICIs-related nephrotic syndrome

**Figure 6.**
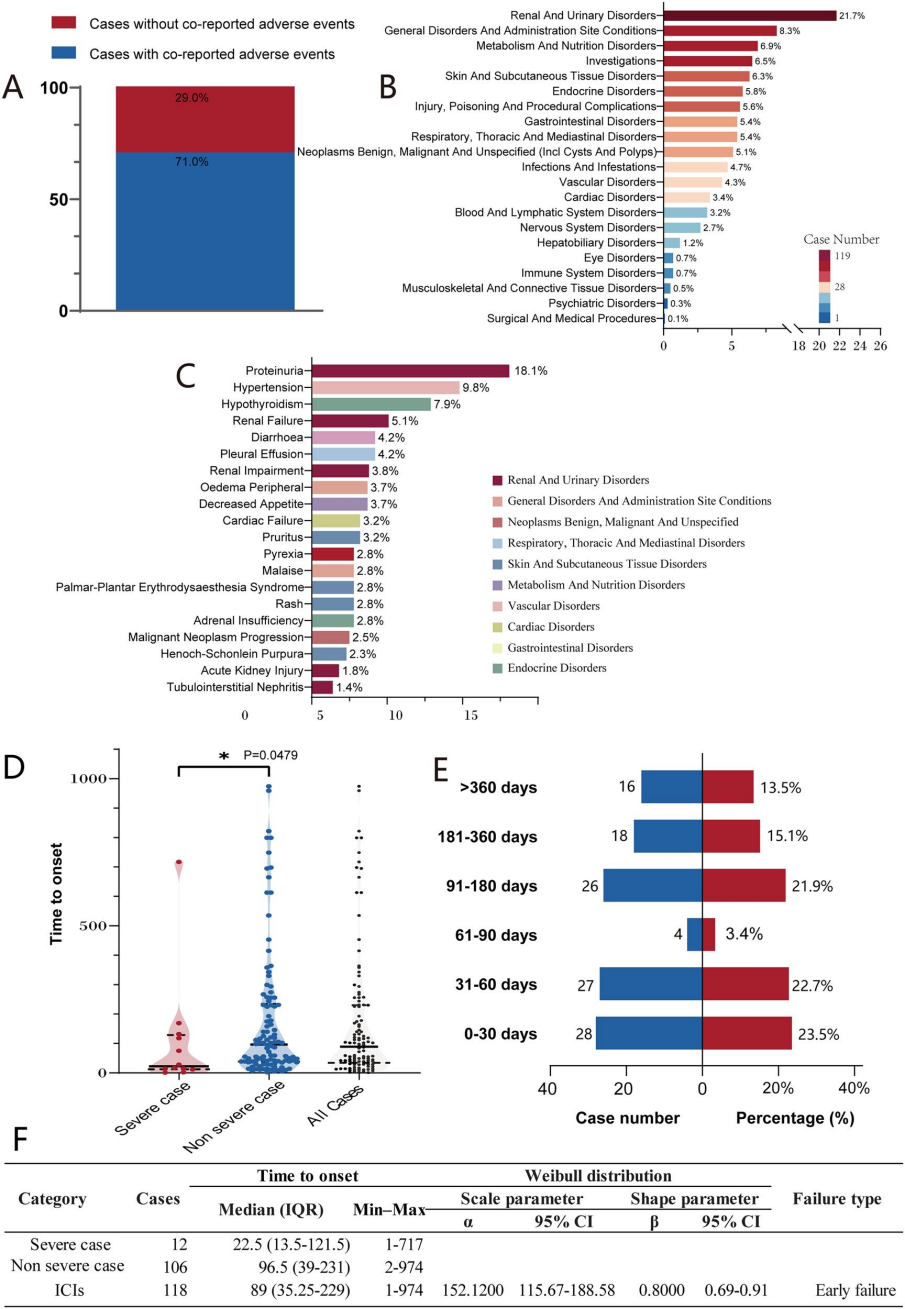
Characteristics of co-reported adverse events and time to onset for ICI-related nephrotic syndrome (A) proportions of cases with or without co-reported adverse events. (B) Most frequent SOCs of co-reported adverse events. (C) Top 20 co-reported adverse events at the PT level. (D) Violin plot of time to onset in serious vs. non-serious cases. P-value is from the Mann-Whitney U test. (E) Frequency distribution of time to onset. (F) Summary of weibull distribution analysis for time to onset.

We examined adverse events that were co-reported with nephrotic syndrome. Among the 404 eligible FAERS reports, 71.0% listed at least one additional adverse event (Figure 6A). At the System Organ Class level, the five most frequently cited categories were: Renal and urinary disorders (21.7%), General disorders and administration-site conditions (8.3%), Metabolism and nutrition disorders (6.9%), Investigations (6.5%) and Skin and subcutaneous tissue disorders (6.3%) (Figure 6B). At the Preferred Term level, the single most common adverse event was proteinuria (18.1%), followed—in descending order—by hypertension (9.8%), hypothyroidism (7.9%), renal failure (5.1%) and diarrhea (4.2%) (Figure 6C).

In FAERS, the median onset time of nephrotic syndrome associated with ICIs was 89 days (IQR 35.25–229), and nearly 45% of cases developed within the first two months of therapy. Severe cases developed significantly earlier than non-severe ones: median 22.5 days (IQR 12.5–121.5) vs 96.5 days (IQR 39–231), respectively (*p* = 0.0479). The Weibull shape parameter β and its 95% CI were both < 1, indicating an early-failure pattern in which the hazard of nephrotic syndrome declines over time (Figure 6D-6F).

### Case series

Our study ultimately included 16 publications encompassing 18 patients. Most were male (*n* = 15, 83.3%) with a median age of 63.5 years (IQR 56.3–69.8 years) (Table 2). 15 events (83.3%) arose during anti-PD-1 therapy, whereas single cases were linked to an anti-PD-L1 antibody, an anti-CTLA-4 antibody, and a combination of nivolumab and ipilimumab (each 1/18, 5.6%). The leading oncologic indications were lung adenocarcinoma (*n* = 4, 22.2%) and melanoma (*n* = 3, 16.7%); the remainder involved renal cell carcinoma, malignant pleural mesothelioma, Hodgkin lymphoma and other tumors. Time to onset ranged from 10 days to 18 months after the first ICI dose.

Renal biopsy most commonly showed MCD (*n* = 8, 44.4%), followed by MN (*n* = 6, 33.3%), FSGS (*n* = 3, 16.6%), and MPGN (*n* = 1, 5.5%) (Supplementary Table S11). Of the 18 patients, 15 were treated with steroids—3 of whom also received an additional immunosuppressive agent—whereas the remaining 3 patients received supportive care only. Among the 15 steroid-treated patients, 12 (80.0%) achieved complete or partial remission, whereas three showed no meaningful response. Eight patients (44.4%) ultimately died, all from tumor progression or complications of underlying disease; five had already achieved remission of nephrotic syndrome at the time of death. ICIs were re-challenged in four patients, and nephrotic syndrome recurred in three. The only relapse-free patient received concomitant steroids during the rechallenge. In addition, one patient relapsed while tapering steroids, but improved again after the steroid dose was increased.

## Discussion

Continuous post-marketing monitoring and evaluation of drug safety constitutes the cornerstone of any pharmacovigilance system [40]. Although ICIs have been widely used in clinical practice for years, knowledge of their long-term and rare adverse events remains incomplete. This study is the first pharmacovigilance investigation of ICI-related nephrotic syndrome based on real-world data from the FAERS database. We identified the associations between different ICI regimens and nephrotic syndrome, characterized the clinical features of the reported cases, and employed Firth multivariable logistic regression to explore potential risk factors.

### Baseline data description

Although the 404 reported cases remain rare among millions of adverse event reports, we observed a clear year-on-year increase in both the number of such reports and their proportion among all ICI-related adverse events. On the one hand, ICI–related nephrotic syndrome is a rare adverse event that has only begun to emerge with the widespread adoption of these agents; for instance, the first published case of pembrolizumab-related nephrotic syndrome appeared in 2016, two years after the drug received regulatory approval [16,41]. On the other hand, this trend likely reflects a growing clinical awareness of this adverse effect; in the earlier stages, underreporting or misdiagnosis may have occurred due to limited recognition. These findings suggest that nephrotic syndrome secondary to ICI therapy has become a clinically significant concern. Therefore, in the context of ICI-based treatment, renal toxicity—particularly severe immune-related glomerular diseases that may lead to nephrotic syndrome—should be incorporated into routine monitoring protocols and considered in differential diagnoses.

### Disproportionality signal

Our study identified a significant association between various ICI therapies and the occurrence of nephrotic syndrome, with the most prominent signal observed for the PD-L1 inhibitor atezolizumab (ROR 6.84). Positive signals were also detected for the most widely used PD-1 inhibitors (nivolumab and pembrolizumab), the CTLA-4 inhibitor ipilimumab in monotherapy, the nivolumab–ipilimumab combination regimen, as well as for two PD-L1 inhibitors—avelumab and durvalumab—for which no prior reports of nephrotic syndrome had been documented in the literature. In contrast, while cemiplimab and dostarlimab did not generate a positive signal, this should not be interpreted as an absence of risk. Given that the data for these two agents are relatively limited or their time on the market is shorter [42], we cannot definitively conclude that they are not associated with a risk of nephrotic syndrome. Continued monitoring of long-term data for these drugs is therefore warranted.

Previous studies have shown that anti-CTLA-4 therapy, whether administered alone or in combination regimens, poses a greater risk of nephrotoxicity than PD-1 inhibitor monotherapy [11,43–46]. While pharmacovigilance databases cannot be used to directly compare the absolute incidence or risk between drugs, we have observed over the past several years that both the risk signal (measured by ROR) and the reporting proportion of nephrotic syndrome associated with PD-1 inhibitors are higher than those for CTLA-4 inhibitors. This is corroborated by our literature review, which identified only two reported cases of nephrotic syndrome induced by ipilimumab, either as monotherapy or in combination therapy [16,31]. This phenomenon suggests that the risk estimation for nephrotic syndrome may differ from the conclusions of prior research. However, this finding does not contradict previous conclusions; rather, it likely reflects the pathophysiological heterogeneity of ICI-induced renal injury. Most clinical trials report renal injury based on laboratory findings, with pathological diagnoses often being absent. Consequently, these studies do not typically discriminate between AIN and glomerular diseases when assessing nephrotoxicity [43–46]. Given that AIN is the predominant pathology in ICI-related acute kidney injury [10], previous conclusions may primarily reflect the risk of AIN while overlooking the distinct risk profile of nephrotic syndrome. As a form of glomerular disease with diverse pathological types [47], nephrotic syndrome likely involves more complex induction mechanisms by ICIs than those underlying AIN.

The regulatory function of CTLA-4 in the early phases of T-cell activation is probably the reason for the increased risk of AIN linked to anti-CTLA-4 agents as opposed to PD-1 inhibitors [48]. Because it acts at an upstream position within the T-cell activation cascade, its blockade has a more widespread impact, thereby increasing the likelihood of tubulointerstitial injury. However, when the injury manifests as nephrotic syndrome, the mechanisms become more complex and diverse. Among these, the most common pathological type is MCD, which is essentially a pattern of podocyte injury. Regarding the mechanism, Shimada et al. proposed a “two-hit” theory that included the induction of CD80 and regulatory T-cell dysfunction [49]. CTLA-4, a costimulatory receptor, downregulates CD80 expression on antigen-presenting cells [50]. When this pathway is blocked by anti‑CTLA‑4 agents, the natural “clearance” effect is impaired, which can lead to podocyte injury and increased CD80 levels. The PD-1/PD-L1 pathway restrains T-cell activation while promoting the differentiation and maintenance of regulatory T cells through inhibition of Akt/mTOR signaling [51]. Consequently, anti-PD-1 therapy can disrupt this regulatory axis, potentially leading to Treg dysfunction and subsequent podocyte injury. Beyond podocytopathy, nephrotic syndrome can also arise from immune complex deposition, a phenomenon more specifically linked to anti-PD-1 inhibition. PD-1 deficient mice have been reported to exhibit several characteristic phenotypes, including a selective increase in serum IgG3 and elevated levels of IgG3 antibodies induced by T-cell independent type 2 antigens [52]. IgG is the primary constituent of nephritogenic immune complexes. In line with it, in the BXSB mouse model of spontaneous lupus nephritis, enhancing PD-1 pathway signaling *via* PD-L1 gene transfer effectively reduced glomerular IgG deposition and ameliorated nephritis [53]. Thus, the presence of PD-1 inhibitors is likely to contribute to the progression of immune complex-mediated kidney disease to some degree. Notably, Abhijat Kitchlu et al. similarly note that anti-CTLA-4 agents did not demonstrate a higher risk than PD-1/PD-L1 inhibitors within the scope of glomerular disease [54]. In conclusion, our results offer a new perspective on the complexity of ICI-related nephrotoxicity. However, considering the current lack of a systematic understanding of its underlying mechanisms, these conclusions must be interpreted with caution. Further research is needed to validate these results.

### Risk factors

Using Firth logistic regression, we identified independent risk factors for ICI-related nephrotic syndrome. People over 65 years old were much more likely to get nephrotic syndrome, underscoring the need for vigilant monitoring in this population. Across tumor types, the risk in patients with renal cancer and mesothelioma was markedly greater than that in patients with lung cancer (aOR = 1.51 and 7.35, respectively). The finding that renal cancer is an independent determinant is concordant with previous reports [45,55]. Due to the limited self-replication capacity of adult podocytes, nephrectomy-induced reduction in nephron number increases filtration pressure on remaining normal podocytes. When compensatory podocyte hypertrophy reaches its limit, focal foot process detachment and podocyte loss occur, ultimately leading to the development of adaptive focal segmental glomerulosclerosis [56,57]. Furthermore, the inherent high responsiveness of renal cell carcinoma to ICI therapy may also contribute to an increased risk of renal injury [58,59]. Importantly, our study is the first to implicate mesothelioma as an independent risk factor for ICI-related nephrotic syndrome. Although mesothelioma-associated nephrotic syndrome is uncommon, several cases have been documented, most presenting with membranous nephropathy or minimal-change disease; in some instances, the syndrome developed even before the initiation of cytotoxic chemotherapy [60–64]. These observations indicate that mesothelioma-associated paraneoplastic immune mechanisms may partially initiate or exacerbate renal injury [65]. Consequently, mesothelioma should be considered an independent, high-priority risk factor during ICI therapy, and affected patients should undergo proactive, prospective risk management. Concomitant administration of the anti-angiogenic agents bevacizumab (aOR = 11.07) and lenvatinib (aOR = 3.00) also emerged as independent predictors of nephrotic syndrome, consistent with their ability to induce glomerular endothelial injury and proteinuria through vascular endothelial growth factor pathway inhibition [66]. In the normal kidney, podocytes constitutively express and secrete VEGF, which acts on VEGF receptors located on glomerular and peritubular endothelium and mesangial cells to maintain glomerular function and the integrity of the glomerular basement membrane. Beyond nephrotic syndrome, VEGF inhibitors can also lead to thrombotic microangiopathy and hypertension. Given that, in clinical practice, immune checkpoint inhibitors combined with anti-angiogenic agents have become standard regimens for advanced hepatocellular carcinoma, renal cell carcinoma, and endometrial cancer [67–72], this strategy often offers superior tumor control but warrants heightened vigilance for nephrotoxicity. Our findings underscore the importance of dynamic monitoring of renal function during such therapy.

### Time to onset analysis

In terms of onset time, the median onset time for ICI-related nephrotic syndrome in the FAERS database was 89 days (IQR:35.25–229). Its onset is often late and highly variable. This timing characteristic is similar to that reported for other ICI-related kidney injuries [11,73]. It is worth noting that severe cases had a shorter median onset time than non-severe cases (22.5 vs. 96.5 days, *p* < 0.05), which suggests that disease progression is more rapid in severe cases. A plausible explanation is that severe cases may have a poorer baseline health status or preexisting subclinical renal dysfunction, which predisposes them to drug-related side effects. This underscores the need for heightened vigilance regarding renal-related symptoms occurring early in the treatment process. The WSP test showed that the upper limit of the 95%CI for the *β* was less than 1, which means that the risk of this adverse event slowly went down over time. However, considering the typically long treatment cycles in cancer patients, even though the risk may decrease over time, the cumulative risk persists. Therefore, ongoing monitoring is crucial, and its frequency should be adjusted based on the clinical context. While routine pre-dosing screening (serum creatinine and urinalysis before each cycle) is fundamental for assessing cumulative safety, it may be insufficient for the early detection of acute toxicity, particularly in patients on extended-interval dosing regimens. For these patients, clinicians should consider interim monitoring, such as a checkup 2–4 weeks post-infusion, to enable the timely detection and management of acute adverse events.

### Clinical management (based on literature review)

Current guidelines for the management of ICI related AIN recommend a severity-stratified approach [12,13,74,75]. For Grade 1–2 AKI, treatment is initiated with oral prednisone at 0.8–1.0 mg/kg/day, whereas for Grade 3 AKI, a short course of intravenous pulse methylprednisolone (0.25–1 g/day for 1–3 days) is advised. This is followed by a meticulous taper over 6–10 weeks, for instance, by reducing the dose by 10 mg weekly down to 20 mg, and then by 5 mg weekly. In contrast, standardized guidance for glomerular disease is lacking. In our literature review, the vast majority of patients (83.3%) received a glucocorticoid-based regimen and achieved significant clinical remission (80%), providing evidence to support glucocorticoids as a first-line therapy. However, the optimal steroid dosage for glomerular disease still requires investigation through prospective cohort studies. The clinical management of ICI-related nephrotic syndrome is often more challenging than that of AIN, necessitating more complex immunosuppressive strategies. Our data corroborate this, as some patients responded favorably to rituximab or mycophenolate mofetil as second-line or adjunctive therapies [18,25,30]. This underscores the necessity of tailoring treatment to the specific type of glomerular pathology. Therefore, a timely kidney biopsy is crucial for determining the underlying pathology and guiding appropriate therapy. This comprehensive management pathway is outlined in our proposed clinical algorithm (Supplementary Figure S1). It should be noted that given the limited and largely retrospective nature of the available cases, this algorithm is not intended as a definitive protocol but rather as a proposed framework informed by our findings and existing irAE management guidelines [12,13,74,75]. It is noteworthy that while most patients had favorable renal outcomes, several ultimately succumbed to the progression of their primary malignancy or its complications [17,18,22–24,28,30]. Although this is insufficient to establish this renal complication itself as an independent risk factor for mortality, it profoundly reflects the clinical predicament and challenges of concurrently managing rare and severe immune-mediated renal injuries during oncologic treatment.

### Considerations for ICI rechallenge (based on literature review)

For some patients, the lack of effective alternative therapies may leave re-exposure to ICI-based immunotherapy as the only viable option. Recurrence is a primary challenge associated with ICI re-administration. A study by Busra Isik et al. included 37 patients with ICI-related acute kidney injury, of whom 16 (43%) underwent a re-challenge, with 3 (19%) experiencing acute kidney injury recurrence [76]. In a multicenter study by Frank B Cortazar et al. 31 of 138 patients with ICI-AKI were re-challenged with an ICI, and 23% of them developed recurrent ICI-AKI [43]. The largest study on ICI-AKI to date, which enrolled 429 patients, reported that 121 (28.2%) were re-challenged. Among these 121 patients, 42 (34.7%) had an initial Grade 3 ICI-AKI, yet the recurrence rate of ICI-AKI upon re-challenge was only 16% [77]. In our case analysis, however, re-challenge was reported in only four patients, three of whom exhibited a recurrence of glomerular disease [17,20,30]. Due to the small number of cases, no definitive conclusion can be drawn from this discrepancy. Notably, while one patient received concomitant steroids during the ICI re-challenge [21], there is currently insufficient data to support the prophylactic use of low-dose steroids during ICI re-administration. Under present guidelines, the decision to use corticosteroids with an ICI rechallenge depends on the severity of the initial ICI-related AKI and the presence of other extra-renal irAEs [12,13]. In summary, ICIs rechallenge is an individualized therapeutic decision. For patients who demonstrate a favorable response to initial treatment (i.e. serum creatinine and/or proteinuria returning to near baseline) and lack effective alternative therapies, ICIs rechallenge may still be a potentially life-saving option, provided that strict monitoring and proactive management are implemented.

### Limitations and future perspectives

Our study has several limitations that highlight critical areas for future investigation. First, there is an inherent limitation in using disproportionality analysis, as a positive signal does not establish causality, and findings could be compromised by unknown confounders. Second, the FAERS database relies on spontaneous, voluntary reporting, a system inherently subject to under-reporting. This means the frequency of reported events should be interpreted as a signal of potential risk rather than a precise measure of real-world incidence. This issue is compounded by classification errors at the Preferred Term level (e.g. reporting “nephrotic syndrome” without pathological subtyping), which weakens statistical power. Third, our regression models were constrained by missing data, such as age, indication, and clinical details like drug dosage, precluding a more in-depth analysis of risk factors. Collectively, these limitations underscore the urgent need for prospective, multicenter cohort studies to definitively validate these safety signals. Such studies are crucial for addressing key clinical gaps, including defining an optimal monitoring strategy (e.g. clarifying proteinuria thresholds for biopsy) and evaluating the efficacy and safety of second-line immunosuppressive agents as adjunctive therapy for steroid-refractory or complex cases. Another critical area is determining the safety and efficacy of ICI rechallenge under prophylactic immunosuppression. Ultimately, incorporating translational research is essential. While some potential biomarkers have been identified for ICI-related interstitial nephritis, robust biomarkers for predicting or monitoring ICI-associated glomerular diseases are notably lacking [78,79]. Investigating novel approaches, such as urinary proteomics or tissue-based PD-1/PD-L1 immune signatures, offers a promising avenue for risk stratification and personalized treatment, improving the care of patients receiving immunotherapy.

In summary, we found that ICIs are broadly associated with a disproportional reporting signal for nephrotic syndrome. Advanced age, renal malignancy, malignant mesothelioma, and concurrent use of bevacizumab or lenvatinib were identified as potential risk factors. The onset of this toxicity was typically early, occurring even sooner in severe cases. A literature review revealed that minimal change disease and membranous nephropathy are the predominant pathological findings, with most cases responding favorably to glucocorticoids. These findings can aid clinicians in the timely identification and management of nephrotic syndrome, thereby improving treatment durability and patient quality of life.

## Supplementary Material

Supplementary Table.docx

Supplementary Figure S1.tif

## Data Availability

The original contributions presented in the study are included in the article. All supplementary tables are available in the Zenodo repository, DOI: 10.5281/zenodo.17156059. Further inquiries can be directed to the corresponding author.
