# Supplementary material for: Immune checkpoint inhibitor-induced nephrotic syndrome: a pharmacovigilance analysis of 404 FAERS cases and literature case series
Source: Ren Fail. 2025 Oct 14;47(1):2569089. doi: 10.1080/0886022X.2025.2569089 (PMC12523462; doi:10.1080/0886022X.2025.2569089)
Supplement: Supplementary Table.docx [file IRNF_A_2569089_SM4662.docx]

**Supplementary Table S1** Methodology of disproportionality analyses.

A Fourfold table of disproportionality analyses.

| Medicine | Target adverse events reported | Other adverse events reported | Summation |
| --- | --- | --- | --- |
| Target drugs | a | b | a+b |
| Other drugs | c | d | c + d |
| Summation | a+c | b + d | a+b + c + d |

a, Number of reports containing both ICIs and nephrotic syndrome; b, Number of reports containing ICIs and other adverse events; c, Number of reports containing other drugs and nephrotic syndrome; d, Number of reports containing other drugs andother adverse events.

B ROR method

| Algorithms | Equation |
| --- | --- |
| ROR | ROR = ad/bc 95%CI = eln(ROR)±1.96(1/a+1/b+1/c+1/d) ^ 0.5 |

**Supplementary Table S2 List of the most frequently co-administered drugs with icis in the faers database.**

| **Drug** |
| --- |
| Carboplatin |
| Paclitaxel |
| Bevacizumab |
| Amlodipine |
| Lenvatinib |
| Cisplatin |
| Ondansetron |
| Pantoprazole |
| Cabozantinib |
| Atorvastatin |
| Acetaminophen |
| Etoposide |
| Metformin |
| Fluorouracil |
| Oxycodone |
| Dexamethasone |
| Oxaliplatin |
| Aspirin |
| Prednisone |
| Omeprazole |
| Levothyroxine |
| Bisoprolol |
| Gemcitabine |
| Cyclophosphamide |
| Apixaban |
| Morphine |
| Prednisolone |
| Loperamide |
| Folic |
| Pregabalin |
| Furosemide |
| Cholecalciferol |
| Lansoprazole |
| Axitinib |
| Ibuprofen |
| Lorazepam |
| Metoclopramide |
| Sodium |
| Gabapentin |
| Docetaxel |

**Supplementary Table S3** L**ist of nephrotoxic medications excluded in sensitivity analysis.**

| **Drug** |
| --- |
| Cisplatin |
| Carboplatin |
| Bevacizumab |
| Lenvatinib |
| Cabozantinib |
| Axitinib |
| Cyclophosphamide |
| Pemetrexed |
| Ibuprofen |
| Aspirin |
| Omeprazole |
| Pantoprazole |
| Lansoprazole |
| Furosemide |
| Lithium |
| Vancomycin |
| Acetaminophen |

**Supplementary Table S4 List of** other novel antineoplastic agents **included in this study.**

| **Category** | **Drug** |
| --- | --- |
| BRAF/MEK Inhibitors | Vemurafenib |
|  | Dabrafenib |
|  | Trametinib |
|  | Encorafenib |
| ADCs | Ado-trastuzumab Emtansine |
|  | Enfortumab Vedotin |
|  | Sacituzumab govitecan |
| PARP Inhibitors | Olaparib |
|  | Rucaparib |
|  | Niraparib |
| ALK Inhibitors | Alectinib |
|  | Brigatinib |
|  | Lorlatinib |
|  | Ceritinib |
| MET Inhibitors | Capmatinib |
| EGFR Inhibitors | Osimertinib |
| Androgen Receptor Signaling Inhibitors | Abiraterone Acetate |
|  | Enzalutamide |
| CDK4/6 Inhibitors | Palbociclib |
| TRK Inhibitors | Larotrectinib |
| KRAS G12C Inhibitors | Sotorasib |
| Anti-angiogenic agents | Vandetanib |
|  | Axitinib |
|  | Cabozantinib |
|  | Regorafenib |
|  | Lenvatinib |
|  | Nintedanib |
|  | Tivozanib |
|  | Fruquintinib |

**Supplementary Table S5 Detailed results of sensitivity analyses.**

| **Category** | **Drug** | **Initial signal** | **Corrected for reporter bias** | **Corrected for concomitant medication bias** | **Corrected for indication bias** |
| --- | --- | --- | --- | --- | --- |
| Anti-PD-1 | Nivolumab | 4.37 ( 3.64 - 5.25 ) | 6.99 ( 5.57 - 8.77 ) | 4.54 ( 3.63 - 5.68 ) | 4.37 ( 3.64 - 5.25 ) |
|  | Pembrolizumab | 3.44 ( 2.88 - 4.1 ) | 3.76 ( 2.99 - 4.73 ) | 2.05 ( 1.45 - 2.9 ) | 3.41 ( 2.86 - 4.07 ) |
| Anti-PD-L1 | Atezolizumab | 6.84 ( 5.58 - 8.4 ) | 7.77 ( 6.23 - 9.69 ) | 3.61 ( 2.14 - 6.1 ) | - |
|  | Avelumab | 5.54 ( 2.64 - 11.63 ) | 6.71 ( 3.01 - 14.94 ) | 3.47 ( 0.87 - 13.87 ) | - |
|  | Durvalumab | 2.03 ( 1.12 - 3.66 ) | 2.46 ( 1.23 - 4.92 ) | 2.1 ( 1 - 4.41 ) | 2.03 ( 1.12 - 3.67 ) |
| Anti-CTLA-4 | Ipilimumab | 2.05 ( 1.07 - 3.94 ) | 3.81 ( 1.43 - 10.17 ) | 2.15 ( 1.07 - 4.29 ) | 1.83 ( 0.91 - 3.66 ) |
| Nivolumab+Ipilimumab | Nivolumab+Ipilimumab | 4.56 ( 3.56 - 5.83 ) | 4.64 ( 3.28 - 6.57 ) | 5.38 ( 4.08 - 7.11 ) | 4.42 ( 3.44 - 5.67 ) |

**Supplementary Table S6** D**isproportionality analysis results for** other novel antineoplastic agents.

| **Category** | **Drug** | **n** | **ROR (95%CI)** |
| --- | --- | --- | --- |
| BRAF/MEK Inhibitors | Vemurafenib | 3 | 0.48 (0.15-1.49) |
|  | Dabrafenib | 9 | 0.84 (0.44-1.62) |
|  | Trametinib | 10 | 0.84 (0.45-1.55) |
|  | Encorafenib | 4 | 1.03 (0.39-2.75) |
| ADCs | Ado-trastuzumab Emtansine | 7 | 1.07 (0.51-2.24) |
|  | Enfortumab Vedotin | 2 | 0.9 (0.22-3.6) |
|  | Sacituzumab govitecan | 0 | 0 (0-0) |
| PARP Inhibitors | Olaparib | 3 | 0.4 (0.13-1.25) |
|  | Rucaparib | 0 | 0 (0-0) |
|  | Niraparib | 0 | 0 (0-0) |
| ALK Inhibitors | Alectinib | 5 | 1.44 (0.6-3.46) |
|  | Brigatinib | 1 | 0.59 (0.08-4.18) |
|  | Lorlatinib | 22 | 8.61 (5.67-13.09) |
|  | Ceritinib | 0 | 0 (0-0) |
| MET Inhibitors | Capmatinib | 0 | 0 (0-0) |
| EGFR Inhibitors | Osimertinib | 9 | 0.93 (0.48-1.79) |
| Androgen Receptor Signaling Inhibitors | Abiraterone Acetate | 4 | 1.03 (0.39-2.75) |
|  | Enzalutamide | 6 | 0.22 (0.1-0.49) |
| CDK4/6 Inhibitors | Palbociclib | 1 | 0.02 (0-0.14) |
| TRK Inhibitors | Larotrectinib | 0 | 0 (0-0) |
| KRAS G12C Inhibitors | Sotorasib | 0 | 0 (0-0) |
| Anti-angiogenic agents | Vandetanib | 1 | 0.88 (0.12-6.26) |
|  | Axitinib | 49 | 5.38 (4.06-7.12) |
|  | Cabozantinib | 67 | 2.47 (1.94-3.14) |
|  | Regorafenib | 27 | 3.13 (2.14-4.56) |
|  | Lenvatinib | 151 | 8.28 (7.04-9.72) |
|  | Nintedanib | 34 | 1.76 (1.26-2.47) |
|  | Tivozanib | 0 | 0 (0-0) |
|  | Fruquintinib | 15 | 14.34 (8.63-23.81) |

**Supplementary Table S7** R**esults of firth univariate logistic regression for ici-related nephrotic syndrome.**

| **Variable** | **P_Value** | **OR (95%CI)** |
| --- | --- | --- |
| **Age** |  |  |
| <65 |  | 1 (Reference) |
| >65 | 0.0001 | 1.6348 (1.2772-2.107) |
| **Sex** |  |  |
| Female |  | 1 (Reference) |
| Male | 0.0278 | 1.3187 (1.0302-1.6996) |
| **Cancer Type** |  |  |
| Malignant Pulmonary Neoplasm |  | 1 (Reference) |
| Malignant Hepatic Neoplasm | 0.0023 | 2.067 (1.3106-3.1534) |
| Malignant Cervical Neoplasm | 0.0644 | 2.5286 (0.9381-5.4679) |
| Aizhenghebing | 0.1802 | 1.9104 (0.709-4.1286) |
| Cutaneous Melanoma | 0.039 | 0.6339 (0.3959-0.9779) |
| Hodgkin Lymphoma | 0.1817 | 2.0493 (0.6756-4.7269) |
| Malignant Mesothelioma | <0.0001 | 8.3271 (5.0457-13.1499) |
| Malignant Thyroid Neoplasm | 0.3482 | 2.4205 (0.2748-8.8304) |
| Malignant Colorectal And Anal Neoplasm | 0.5687 | 0.743 (0.2061-1.8704) |
| Lip And Oral Cavity Neoplasms Malignant | 0.1425 | 3.0268 (0.6288-8.736) |
| Malignant Ovarian Neoplasm | 0.3625 | 1.6042 (0.529-3.6984) |
| Malignant Bladder Neoplasm | 0.8647 | 1.0872 (0.3586-2.5051) |
| Skin Neoplasms Malignant (Excl Melanoma) | 0.744 | 1.2423 (0.2584-3.5752) |
| Other Malignant Neoplasms | 0.1994 | 1.7611 (0.713-3.6194) |
| Malignant Prostate Neoplasm | 0.5857 | 0.6576 (0.0748-2.3891) |
| Breast Neoplasms Malignant | 0.4144 | 0.7636 (0.3646-1.4174) |
| Malignant Renal Neoplasm | 0.002 | 1.7626 (1.2358-2.4885) |
| Malignant Esophageal Neoplasm | 0.1721 | 0.4619 (0.0961-1.3275) |
| Malignant Head And Neck Neoplasm | 0.4126 | 0.5435 (0.0618-1.9742) |
| Malignant Gastric Neoplasm | 0.4883 | 0.7465 (0.2773-1.6116) |
| Malignant Pancreatic Neoplasm | 0.7267 | 1.2173 (0.3375-3.0664) |
| Malignant Endometrial Neoplasm | 0.1277 | 1.5805 (0.8684-2.6727) |
| **Carboplatin** |  |  |
| No |  | 1 (Reference) |
| Yes | 0.0989 | 0.7532 (0.5232-1.0525) |
| **Paclitaxe** |  |  |
| No |  | 1 (Reference) |
| Yes | 0.2643 | 1.247 (0.8389-1.7867) |
| **Bevacizumab** |  |  |
| No |  | 1 (Reference) |
| Yes | <0.0001 | 2.3288 (1.6312-3.2334) |
| **Pemetrexed** |  |  |
| No |  | 1 (Reference) |
| Yes | 0.3783 | 0.8051 (0.4702-1.2814) |
| **Lenvatinib** |  |  |
| No |  | 1 (Reference) |
| Yes | <0.0001 | 2.2429 (1.5775-3.104) |

**Supplementary Table S8** Multicollinearity diagnostics results.

| **Variable** | **GVIF** | **Df** | **GVIF^(1/(2*Df))** |
| --- | --- | --- | --- |
| **Age** | 1.06550748521456 | 1 | 1.03223422013347 |
| **Sex** | 1.38412923329128 | 1 | 1.17649021810267 |
| **Cancer Type** | 3.59660657703392 | 21 | 1.03094511720599 |
| **Bevacizumab** | 1.60447649471881 | 1 | 1.26667931802758 |
| **Lenvatinib** | 1.63341169292041 | 1 | 1.27804995713016 |

**Supplementary Table S9** Bootstrap analysis results.

| **Variable** | **Bootstrap 95% CI** |
| --- | --- |
| **Age** |  |
| <65 | 1 (Reference) |
| >65 | 1.52 (1.18-2.02) |
| **Sex** |  |
| Female | 1 (Reference) |
| Male | 1.31 (0.99-1.78) |
| **Cancer Type** |  |
| Malignant Pulmonary Neoplasm | 1 (Reference) |
| Malignant Hepatic Neoplasm | 1.13 (0.66-1.81) |
| Malignant Cervical Neoplasm | 2.62 (0.74-5.75) |
| Aizhenghebing | 1.67 (0.44-3.31) |
| Cutaneous Melanoma | 0.71 (0.42-1.06) |
| Hodgkin Lymphoma | 2.61 (0.79-5.31) |
| Malignant Mesothelioma | 7.35 (4.09-11.47) |
| Malignant Thyroid Neoplasm | 1.95 (0.53-5.46) |
| Malignant Colorectal And Anal Neoplasm | 0.74 (0.10-1.61) |
| Lip And Oral Cavity Neoplasms Malignant | 3.20 (0.58-7.11) |
| Malignant Ovarian Neoplasm | 1.42 (0.33-3.24) |
| Malignant Bladder Neoplasm | 1.06 (0.32-2.14) |
| Skin Neoplasms Malignant (Excl Melanoma) | 1.23 (0.22-2.92) |
| Other Malignant Neoplasms | 1.84 (0.67-3.46) |
| Malignant Prostate Neoplasm | 0.58 (0.16-1.56) |
| Breast Neoplasms Malignant | 1.12 (0.48-2.07) |
| Malignant Renal Neoplasm | 1.51 (1.04-2.17) |
| Malignant Esophageal Neoplasm | 0.46 (0.08-1.04) |
| Malignant Head And Neck Neoplasm | 0.52 (0.15-1.35) |
| Malignant Gastric Neoplasm | 0.78 (0.21-1.53) |
| Malignant Pancreatic Neoplasm | 1.31 (0.18-2.91) |
| Malignant Endometrial Neoplasm | 0.91 (0.42-1.71) |
| **Bevacizumab** |  |
| No | 1 (Reference) |
| Yes | 2.24 (1.48-3.42) |
| **Lenvatinib** |  |
| No | 1 (Reference) |
| Yes | 2.50 (1.64-3.58) |

**Supplementary Table S10** E-values and 95% CI Limits.

| **Variable** | **OR** | **CI_Lower** | **CI_Upper** | **P_Value** | **E_value_point** | **E_value_CI** |
| --- | --- | --- | --- | --- | --- | --- |
| **Age** |  |  |  |  |  |  |
| <65 |  |  |  |  |  |  |
| >65 | 1.5239 | 1.1808 | 1.9802 | 0.0011 | 2.4174 | 1.6428 |
| **Cancer Type** |  |  |  |  |  |  |
| Malignant Pulmonary Neoplasm |  |  |  |  |  |  |
| Malignant Mesothelioma | 7.3455 | 4.439 | 11.6368 | <0.0001 | 14.1727 | 8.3461 |
| Malignant Renal Neoplasm | 1.5075 | 1.0309 | 2.1776 | 0.0345 | 2.3822 | 1.2094 |
| **Bevacizumab** |  |  |  |  |  |  |
| No |  |  |  |  |  |  |
| Yes | 2.2427 | 1.4346 | 3.4213 | 0.0005 | 3.9121 | 2.2242 |
| **Lenvatinib** |  |  |  |  |  |  |
| No |  |  |  |  |  |  |
| Yes | 2.4962 | 1.5965 | 3.7962 | 0.0001 | 4.4288 | 2.5724 |

**Supplementary Table S11** D**etailed renal biopsy findings of patients from included case reports**

| **Patient** | **Renal Biopsy** |
| --- | --- |
| 1 | The renal biopsy showed diffuse fusions of the epithelial foot processes on electron microscopy compatible with minimal change disease. Direct immunofluorescence microscopy was negative with no complement or immunoglobulin deposits. |
| 2 | The kidney biopsy showed 22 glomeruli on light microscopy without focal sclerosis or increase inmesangial matrix or cellularity. There was focal tubular injury and mild interstitial fibrosis. Immunofluorescence showed positivelinear basement membrane staining for immunoglobulin G (IgG;1+) and κ (1+) and λ light chains (1+), but staining for IgM, IgA,C3, and C1q was negative. Electron microscopy demonstrateddiffuse foot-process effacement without immune-type electron-dense deposits or tubuloreticular inclusions |
| 3 | The kidney biopsy showed 15 glomeruli on light microscopy, one of which was globally sclerosed. The remaining glomeruli showed no increase in mesangial matrix or cellularity and there were no tubulointerstitial changes.Immunofluorescence demonstrated mesangial staining for IgM(1+), IgA (trace), C3 (1+), and C1q (trace), but no staining for IgG or κ or λ light chains. Electron microscopy showed diffuse foot-process effacement. Glomerular basement membranes appeared normal, and there were no immune-type electron- dense deposits or tubuloreticular inclusions, compatible with findings of MCD |
| 4 | The kidney biopsy showed mostly normal glomeruli. A few glomeruli showed segmental collapse of capillaries with surrounding epithelial hyperplasia and epithelial protein droplets, consistent with early focal and segmental glomerulosclerosis (FSGS) with collapsing features. Immunofluorescence staining for IgG, IgA, IgM, C1q, kappa, and lambda was negative, ruling out underlying immune complex–mediated glomerular disease. Electron microscopy showed podocyte microvillous change with massive foot-process effacement, confirming severe podocyte injury and consistent with FSGS |
| 5 | The kidney biopsy showed 20 glomeruli with no obvious changes on light microscopy. The tubulointerstitium and small arterioles showed no remarkable changes. Immunofluorescence showed the specimen was negative for immunoglobulin G, M, and A, C3, C1q, and κ and λ light chains. Electron microscopy demonstrated diffuse podocyte foot process effacement. The final diagnosis was MCD |
| 6 | The kidney biopsy showed a tubular damage on light microscopy (presumably due to a preexistent hypertensive nephropathy) without signs of interstitial nephritis. Amyloidosis, the presence of immune complexes or complement-mediated glomerulonephritis were ruled out by immunohistochemistry. Electron microscopy showed findings consistent with a minimal change disease |
| 7 | The kidney biopsy showed no detectable histologic abnormalities on microscopic low-power view of Haematoxylin-eosin stained sections, and immunofluorescence microscopy did not reveal any glomerular deposits of complement or immunoglobulins. In contrast, electron microscopy showed minor glomerular abnormalities with partial foot process effacement. |
| 8 | The kidney biopsy showed mild to moderate mesangial widening, mild mesangial hypercellularity, and minimal endocapillary proliferation on light microscopy. Segmental double contours of the glomerular basement membrane were identified, but no large subendothelial deposits or hyaline thrombi were seen. Mild, patchy interstitial inflammation consisting of mononuclear leukocytes was present along with acute tubular injury, but the degree of interstitial inflammation was relatively trivial. On immunofluorescence microscopy, there was C3-dominant staining (2+) with less-intense IgM staining (1+) in the mesangium and scattered along the glomerular capillary loops. Ultrastructural analysis revealed scattered subendothelial and mesangial electron-dense immune deposits with associated glomerular basement membrane reduplication and mesangial cell interposition. The majority of the podocyte foot processes were effaced. |
| 9 | The kidney biopsy showed global sclerosis and segmental adhesions, pathological features consistent with Focal Segmental Glomerulosclerosis (FSGS). |
| 10 | The kidney biopsy showed histopathology most consistent with minimal change disease, although was limited by processing artifact. Light microscopy displayed mildly ischemic glomeruli with periglomerular fibrosis. Electron microscopy revealed marked podocyte effacement, moderate focal ischemic changes, patchy interstitial infiltrate and no dense deposits. |
| 11 | The kidney biopsy showed stiffness in the glomeruli on light microscopy with scattered subepithelially localized immune deposits (Masson stain) containing slightly focal tubular atrophy and interstitial fibrosis, consistent with early MN. Immunofluorescence staining showed granular immunoglobulin G (IgG) deposits, including IgG1, IgG2 and IgG4, uniformly and subepithelially distributed in the glomeruli. The immunofluorescence staining of IgG3 was negative. Electron microscopy showed discrete electron-dense deposits at the subepithelial surface of the glomerular capillary wall, accompanied by effacement of overlying epithelial cell foot processes. Immunohistochemical analyses revealed positive staining for THSD7A along the glomerular basement membrane. |
| 12 | The kidney biopsy showed on light microscopy that of up to 36 glomeruli, 2 exhibited segmental sclerosis with atrophied tubules and fibrosis in the interstitium. Electron microscopy revealed wide effacement of the epithelial cell foot processes. Immunofluorescence microscopy showed no immune complexes or autoantibody deposition. |
| 13 | The kidney biopsy showed 19 glomeruli, one of which showed global glomerulosclerosis. The glomerular basement membrane showed diffuse thickening and spike formation. There was no evidence of acute interstitial nephritis or diabetic nephropathy while we observed mild arteriolosclerosis. Immunofluorescence staining revealed IgG and C3 deposition on the capillary walls. There was no staining for IgA, IgM, C4 and C1q. Electron microscopic examination revealed subepithelial electron-dense deposits adjacent to projections of basement membrane material. These observations led to the diagnosis of MN. |
| 14 | The kidney biopsy showed on light microscopy enlarged glomeruli with subtle basement membrane spikes on silver stain, without mesangial or endocapillary hypercellularity, fibrinoid necrosis, or hyaline thrombi. Mild arterial and arteriolar sclerosis, and mild acute tubular epithelial injury were also present. By immunofluorescence microscopy, there was global granular capillary loop staining for immunoglobulin (Ig) G and C3, as well as kappa and lambda light chain. There was no significant staining for IgA, IgM, or C1q. Electron microscopy (EM) revealed glomerular basement membrane thickening with associated subepithelial electron-dense deposits. Rare segmental mesangial and subendothelial deposits were also present. |
| 15 | The kidney biopsy showed diffuse interstitial infiltration of lymphocytes at lower magnification and diffuse crescent formation (14 of 29 glomeruli with cellular crescents and 8 of 29 with fibrocellular crescents). Tubular atrophy and severe interstitial inflammation were seen around a normal glomerulus. Membrane alterations (spikes and thickening) were not visible by light microscopy. Immunofluorescence revealed linear and partially granular staining of the glomerular capillary loops for IgG (2 +), IgA (1 +), and C3 (1 +). IgG1 and IgG3 were predominantly stained in glomerular capillary loops. Immunofluorescence results were negative for glomerular phospholipase A2 receptor (PLA2R) expression. Electron microscopy revealed electron-dense deposits in the subepithelial and mesangial regions, thickening and irregularity of the GBM, and extensive podocyte foot process effacement. |
| 16 | The kidney biopsy showed diffuse thickening and spike formations of the glomerular basal membrane, with positive PLA2R immunohistochemical staining and subepithelial full-house immunofluorescence pattern. Test for PLA2R antigen expression on the lung tumor was negative. Analysis of PLA2R domain recognition showed an anti-CysR restricted activity. |
| 17 | The kidney biopsy showed optically normal glomeruli on histopathological examination, with positive PLA2R immunohistochemical staining. Immunofluorescence showed extramembranous subepithelial deposits of polytypic IgG and C3. |
| 18 | The kidney biopsy showed on light microscopy 11 glomeruli without focal sclerosis, an increase in the mesangial matrix, or cellularity. The renal interstitium exhibited mild fibrosis, mild leukocytic infiltration, and slight tubulitis, accompanied by medial hypertrophy and fibroblastic intimal thickening in the interlobular and arcuate arteries. An immunofluorescence analysis yielded negative results. Electron microscopy revealed fusion of nearly all podocyte foot processes without any electron-dense deposits. |
