# Supplementary figures and images for: Immune checkpoint inhibitor-induced nephrotic syndrome: a pharmacovigilance analysis of 404 FAERS cases and literature case series
Source: Ren Fail. 2025 Oct 14;47(1):2569089. doi: 10.1080/0886022X.2025.2569089 (PMC12523462; doi:10.1080/0886022X.2025.2569089)

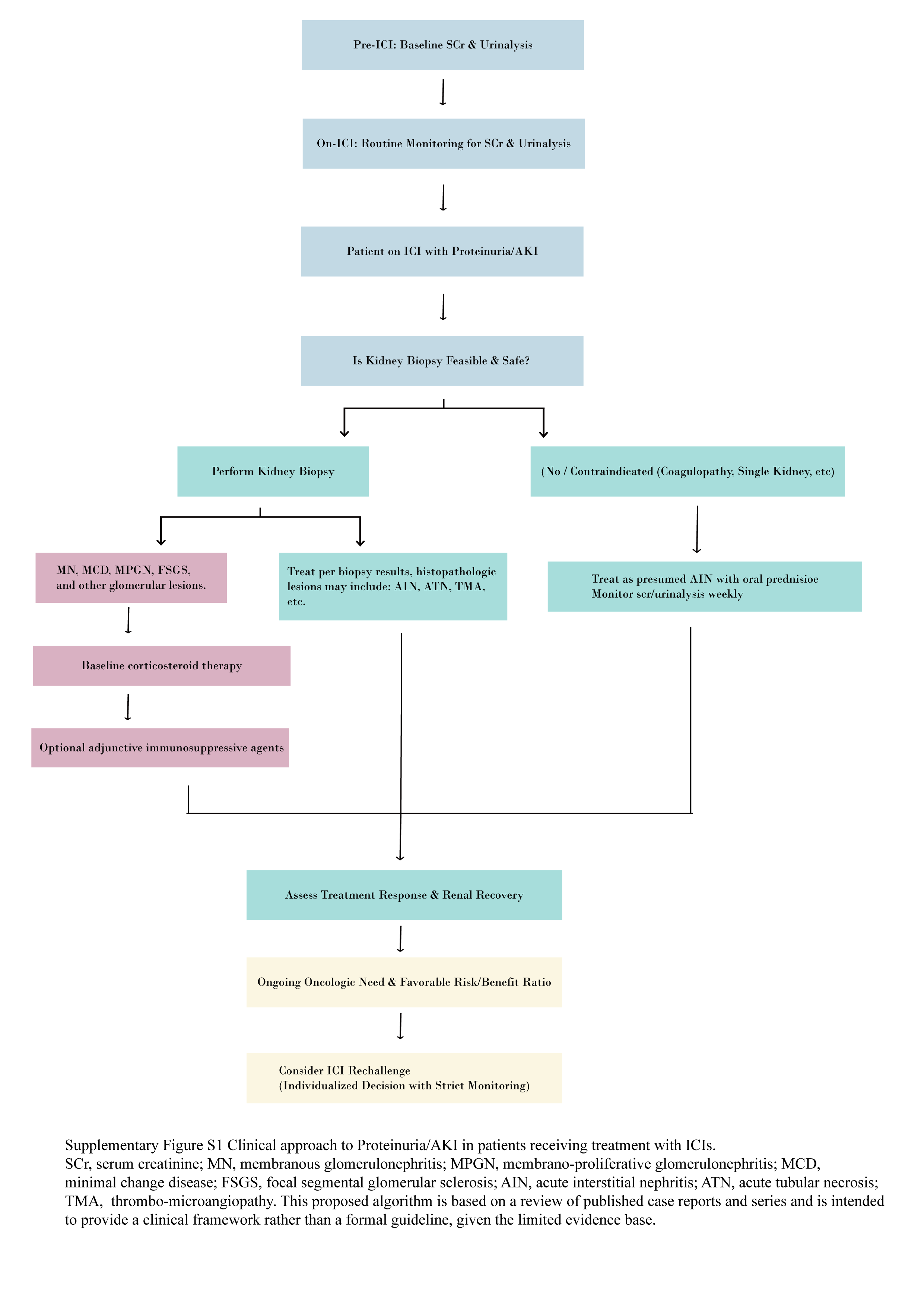

Supplement: Supplementary Figure S1.tif [file IRNF_A_2569089_SM4661.tif]
